# Evidence of Cardiovascular Calcification and Fibrosis in Pseudoxanthoma Elasticum Mouse Models Subjected to DOCA-Salt Hypertension

**DOI:** 10.1038/s41598-019-52808-z

**Published:** 2019-11-08

**Authors:** Loukman Omarjee, Charlotte Roy, Christophe Leboeuf, Julie Favre, Daniel Henrion, Guillaume Mahe, Georges Leftheriotis, Ludovic Martin, Anne Janin, Gilles Kauffenstein

**Affiliations:** 1 MitoVasc Institute, UMR CNRS 6015 - INSERM U1083, Angers University, Angers, France; 2 Univ Rennes, CHU Rennes, INSERM CIC1414, Vascular Medicine Unit, Rennes, France; 3 PXE Vascular Consultation Centre, CHU Rennes, Rennes, France; 4 PXE Reference Centre (MAGEC Nord), University Hospital of Angers, Angers, France; 5 Vascular Medicine Unit, Redon Hospital, 8 Rue Etienne Gascon, Redon, France; 6 Pathology Laboratory, Paris Diderot University, Sorbonne Paris Cité, Paris, France; 7 INSERM U942, Paris, France; 8 Physiology and Vascular Investigation Department, Nice University Hospital, Nice, France; 9 Department of Pathology, Saint-Louis Hospital, APHP, Paris, France

**Keywords:** Peripheral vascular disease, Calcification, Renovascular hypertension

## Abstract

Pseudoxanthoma Elasticum (PXE) is a rare disorder characterized by fragmentation and progressive calcification of elastic fibres in connective tissues. Although arterial hypertension (AHT) has been reported in PXE patients, its impact on pathological manifestations has as yet been unexplored. We investigated the consequences of experimental AHT on *Abcc6*−/− PXE mouse models. Experimental AHT was induced by deoxycorticosterone acetate (DOCA-salt) in uni-nephrectomised mice. Blood pressure (BP) and vascular reactivity were monitored using tail-cuff plethysmography and myography respectively. Calcium content and fibrosis were assessed using colorimetry, Von Kossa and Sirius red staining respectively. The gene expression implicated in vascular biology was measured using quantitative polymerase chain reaction. DOCA-salt induced a matching rise in BP in *Abcc6*−/− and WT mice. Aortic ring contraction and relaxation *in vitro* were comparable. Calcium accumulated in the hearts of hypertensive *Abcc6*−/− mice along with significant fibrosis in the myocardium and aorta by contrast with the WT mice. In hypertensive *Abcc6*−/− mouse aortas, these results were corroborated by gene expression patterns favouring calcification, fibrosis and extracellular matrix remodelling. *Abcc6* loss-of-function is associated with greater cardiovascular calcification and fibrosis in mice subjected to DOCA-Salt hypertension. These results suggest likely cardiovascular deterioration in PXE patients with AHT, necessitating diligent BP monitoring.

## Introduction

Pseudoxanthoma Elasticum (PXE, OMIM 264800) is a genetic disease characterized by fragmentation and progressive calcification of elastic fibers in the skin, eyes and blood vessels^1^. The most common vascular manifestations are peripheral arterial disease (PAD) and increased risk of cerebral infarction^1^. PXE is caused by mutations in the *ABCC6* gene which encodes a transmembrane adenosine triphosphate (ATP)-binding cassette (ABC) transporter primarily expressed in the liver and kidney^2,3^. Several studies have demonstrated that normal tissues exposed to PXE patient serum/plasma tend to calcify, indicating a metabolic etiology underlying this disease consistent with inexistent or low-level ABCC6 transporter expression in affected tissues^4^. No ABCC6 endogenous substrates have as yet been emphatically identified, cause of ectopic calcification^5,6^.

Typical PXE manifestations include yellowish papules on the neck and large skin folds, retinal angioid streaks and cardiovascular (CV) complications^7^ such as: diminished or absent peripheral vascular pulsations; hypertension of unknown origin; echographic opacities due to arterial calcification (especially in the kidneys, spleen and pancreas); AHT; angina pectoris; intermittent claudication (often regarded as the first sign of accelerated atherosclerosis); gastrointestinal hemorrhage; arteriosclerosis; and increased risk of myocardial and cerebral infarction^7^. There is a higher risk of vascular events in PXE patients than in the general population, presumably resulting from arterial medial calcification along with arterial stiffening and thickening^8^. Adverse arterial remodelling and increased aortic stiffness are associated with resistant AHT in the general population^9^. Around 20–25% of PXE patients present CV complications related to high morbidity and mortality such as higher prevalence of accelerated atherosclerosis involving greater risk of complete vascular occlusion^10,11^.

Since *Abcc6*−/− mouse models mimic the majority of human PXE manifestations, their use for research into PXE is warranted^12^. In the present study, *Abcc6−/−* and WT mice were submitted to deoxycorticosterone acetate (DOCA)-salt-induced AHT, representing an experimental model characterized by an initial rise in AHT over the first few days followed by sustained elevated BP for weeks^13^. Our aim was to investigate the consequences of DOCA-salt-induced AHT on CV reactivity and remodelling in *Abcc6*−/− mice by comparison with WT mice.

## Results

### Comparable DOCA-salt induced arterial hypertension and changes in vascular reactivity observed in *Abcc6−/−* and WT mice

**Figure 1:**
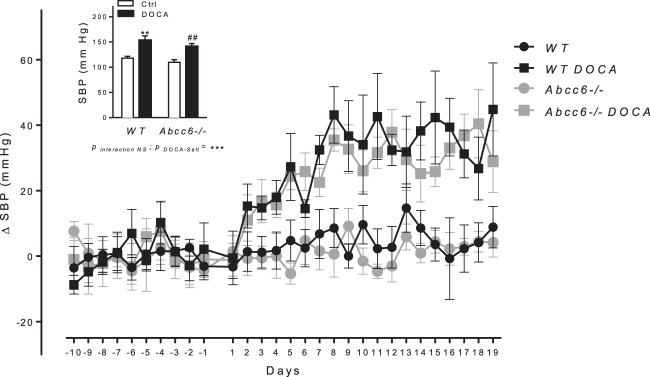
Comparable DOCA-salt-induced hemodynamic parameters changes in *Abcc6*−/− and WT mice. Systolic blood pressure kinetic characteristics as well as mean values over ultimate five days of DOCA-salt treatment. Data represent mean ± SEM of six mice from each group *p < 0.05, **p < 0.01 WT untreated versus DOCA; ^#^p < 0.05, ^##^p < 0.01 *Abcc6*−/− untreated versus DOCA.

**Figure 2:**
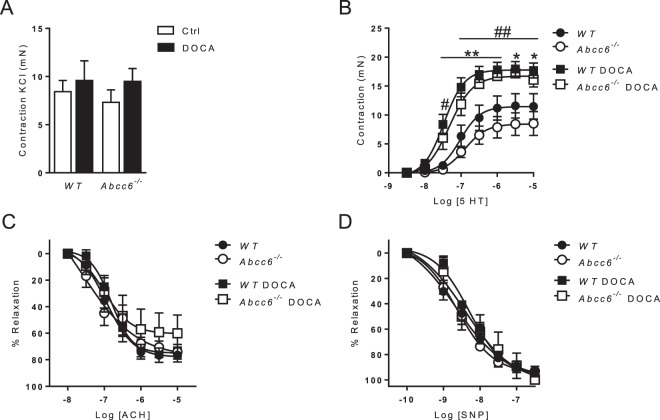
Comparable DOCA-salt-induced vascular reactivity changes in *Abcc6*−/− and WT mice. (**A**) Contraction of aortic rings in response to KCl, (**B**) Dose-response contraction curve in response to 5-HT and (**C**,**D**) endothelium-dependent and -independent relaxation (ACh and SNP, respectively) evaluated using wire myography. Data represent mean ± SEM of six mice from each group *p < 0.05, **p < 0.01 WT untreated versus DOCA; ^#^p < 0.05, ^##^p < 0.01 *Abcc6*−/− untreated versus DOCA.

No difference in resting hemodynamic parameters could be found between *Abcc6*−/− and WT mice (Table 1). DOCA-salt treatment caused a progressive rise in systolic blood pressure (SBP) over a period of 19 days in *Abcc6*−/− and WT mice (Fig. 1). At end of treatment, SBP was 141.4 ± 5.5 mmHg in *Abcc6*−/− DOCA vs 109.7 ± 5.1 mmHg in *Abcc6*−/− mice (p < 0.01) and 153.4 ± 8,8 mmHg in WT DOCA vs 117 ± 3.7 mmHg in WT mice (p < 0.01). There was no difference in SBP between *Abcc6*−/− and WT mice. Vascular contraction and endothelium-dependent and -independent relaxation were measured in the aortic rings using myography. Contractile response to KCl was unaffected by DOCA-salt and was comparable in both mouse strains (Fig. 2A). Contractile response to 5-hydroxytryptamine (5-HT) increased in both *Abcc6*−/− and WT DOCA specimens with no evidence of significant genotype effect (Fig. 2B). Similarly, the pre contractions in response to phenylephrine (1 µM prior Ach) were equivalent according to the genotype (tension in mN: 8.1 ± 4.6 vs 5.4 ± 3.4 for untreated WT and *Abcc6*−/−; 11.6 ± 1.9 vs 11.1 ± 4.3 for DOCA WT vs *Abcc6*−/−), while a significant gain of contraction associated to the DOCA treatment was found (p = 0.007, data not shown). Endothelium-dependent (acetylcholine, ACh) and -independent (sodium nitroprusside, SNP) relaxation was unaffected by DOCA-salt and was comparable in both mouse strains (Fig. 2C,D).

**Table 1:** Hemodynamic parameters of control and hypertensive *Abcc6*−/− and WT mice.

	Pre-treatment		DOCA-salt	
	WT (n = 6)	*Abcc6−/−* (n = 6)	WT (n=6)	*Abcc6−/−* (n=6)
SBP	117.0 ± 3.7	109.7 ± 5.1	153.4 ± 8.8**	141.4 ± 5.5**
DBP	66.0 ± 7.8	61.0 ± 4.5	81.0 ± 9.7	77.0 ± 5.5
MBP	83.2 ± 6.4	77.5 ± 4.8	104.9 ± 9.4	98.4 ± 5.4
PP	52.0 ± 4.9	48.6 ± 1.8	71.8 ± 2.4**	64.4 ± 1.8**
HR	613.3 ± 6.3	568.0 ± 19.3	571.9 ± 24.0	558.8 ± 10.9

SBP, DBP, Mean Blood Pressure (MBP), Pulse Pressure (PP) in mmHg and HR as beats per minute (bpm) of untreated and DOCA-salt-treated mice. Data represent mean ± SEM of six animals from each group. **p < 0.01 DOCA versus control.

### DOCA-salt induced calcium accumulation and fibrosis in *Abcc6−/−* murine hearts

**Figure 3:**
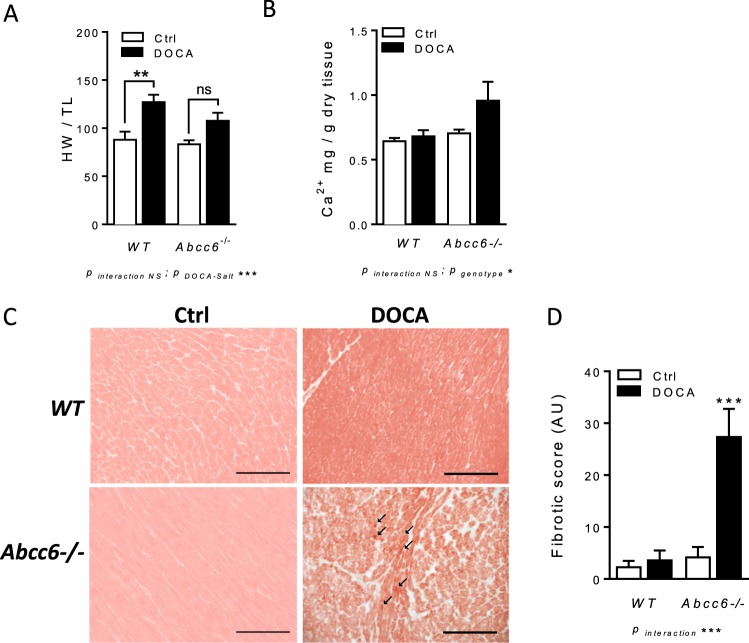
DOCA-salt-induced cardiac remodelling in *Abcc6*−/− mice: hypertrophy, calcium accumulation and fibrosis. (**A**) Heart weight normalized to tibia length (HW/TL) (**B**) Colorimetric determination of total tissue calcium level in left ventricles and (**C**) representative images and (**D**) quantification of Sirius red staining. Data represent mean ± SEM of six mice from each group *p < 0.05, **p < 0.01, ***p < 0.001.

Heart weight to tibia length (HW/TL) ratio was measured to evaluate cardiac hypertrophy, revealing no difference between untreated WT and *Abcc6*−/− mice. DOCA-salt treatment induced a significant increase in HW/TL ratio in WT (88 ± 8 to 127 ± 8; p < 0.01) but not in *Abcc6*−/− mice (Fig. 3A).

Colorimetric quantification of total tissue calcium showed more significant accumulation in the left ventricles (LV) of *Abcc6*−/− DOCA than in *Abcc6*−/− mice (p < 0.05) (Fig. 3B), indicative of dystrophic cardiac calcification (DCC). Comparable tissue calcium increase was not detected in WT mice. All of the Von Kossa staining slides analyzed were negative in the heart LV specimens from the four series of mouse models (data not shown). Heart fibrosis, evidenced by Sirius red staining, occurred in *Abcc6*−/− DOCA but not *Abcc6*−/− mice. This was not observed in WT DOCA as opposed to WT mice (Fig. 3C,D). No significant correlation between heart hypertrophy (HW/TL) and the fibrotic score could be evidenced in both WT DOCA mice and *Abcc6*−/− DOCA mice (data not shown).

### DOCA-salt induced vascular fibrosis as distinct from hypertrophy in *Abcc6−/−* mice

**Figure 4:**
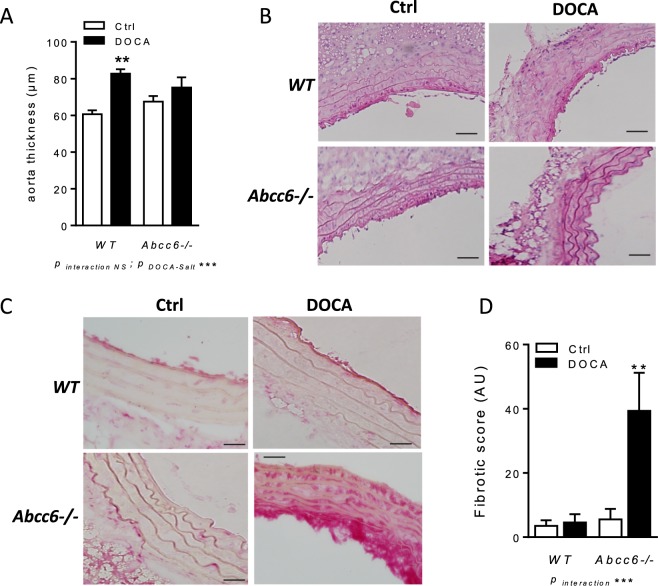
DOCA-salt-induced aortic fibrosis excepting hypertrophy in *Abcc6*−/− mice. (**A**) Wall thickness measured in TA sections. (**B**) Representative images of Von Kossa staining performed on TA sections. (**C**) Representative images and (**D**) quantification of Sirius red staining performed on TA sections. Data represent mean ± SEM of six mice from each group **p < 0.01, ***p < 0.001.

Aortic remodelling arose from hypertension and wall thickness were measured in these animals. Significant vascular hypertrophy was found in WT but not in *Abcc6*−/− DOCA-treated mice (82.7 ± 2.4 µm vs 75.2 ± 5.6 µm; p < 0.05) by comparison with untreated WT and *Abcc6*−/− mice (60.7 ± 2 µm vs 67.5 ± 3.1 µm; p > 0.05) (Fig. 4A). Von Kossa staining was negative in the entire TA slides analyzed (Fig. 4B). Fibrosis was significantly more extensive in *Abcc6*−/− DOCA mice than in *Abcc6*−/− mice. No positive Sirius red staining was recorded in WT DOCA (Fig. 4C,D). No significant correlation between thoracic aorta (TA) hypertrophy and the fibrotic score could be evidenced neither in WT DOCA nor in *Abcc6*−/− DOCA mice (data not shown).

### Molecular approach to vascular remodelling in hypertensive *Abcc6−/−* and WT mice

**Figure 5:**
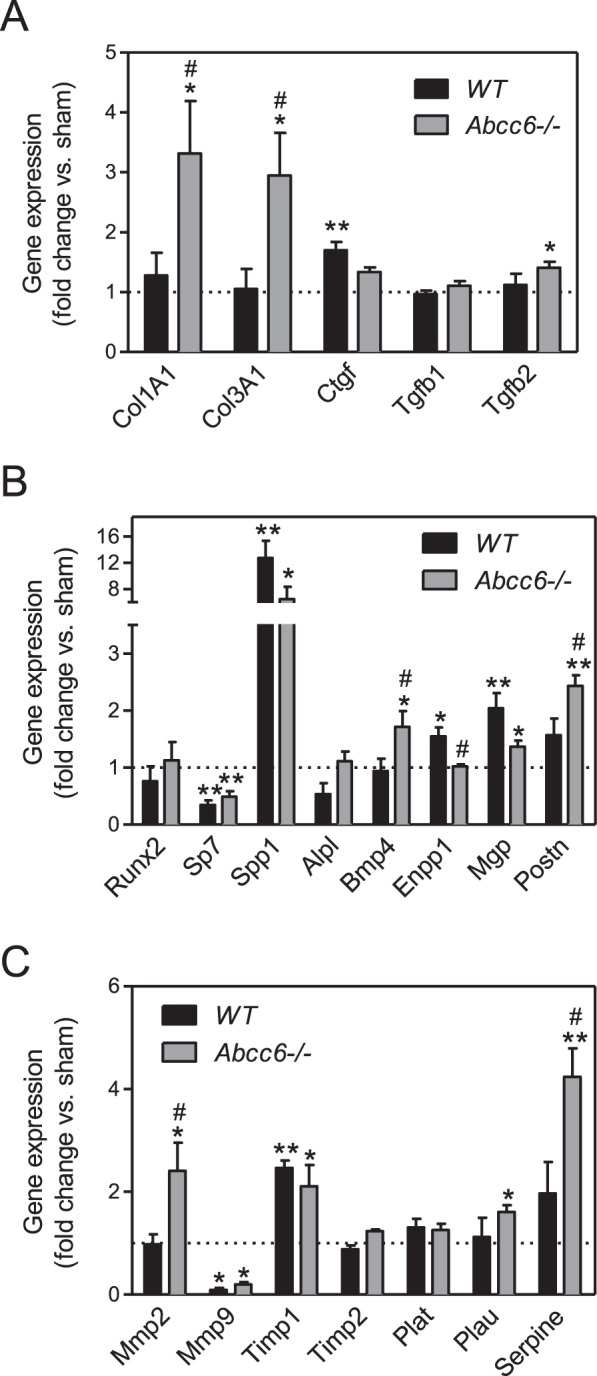
Molecular pattern of vascular remodelling in hypertensive *Abcc6*−/− and WT mice. Gene coding for proteins involved in (**A**) fibrosis, (**B**) ectopic calcification and (**C**) matrix remodelling evaluated by RT-qPCR in the TA. Data represent fold change versus untreated ± SEM from four to six mice per group (Student’s t-test). *p < 0.05, **p < 0.01 untreated versus DOCA. ^#^p < 0.05, ^##^p < 0.01 WT DOCA versus *Abcc6*−/− DOCA.

We evaluated expression of gene coding in the proteins involved in fibrosis (Fig. 5A), ectopic calcification (Fig. 5B) and matrix remodelling (Fig. 5C) in the TA. No statistical differences were found in gene expression levels between untreated *Abcc6*−/− and WT mice. Of the genes involved in the fibrotic process, significant increase in collagen 1 and 3 (*Col1a1* and *Col3a1*) and *Tgfβ*2 expression was found exclusively in *Abcc6*−/− DOCA mice while a significant increase in connective tissue growth factor (*Ctgf*) expression was found exclusively in WT DOCA mice (Fig. 5A). Among the genes involved in ectopic mineralization, transcription factor *Sp7* expression decreased while osteopontin (*Spp1*) and matrix Gla protein (*Mgp*) expression increased in both *Abcc6*−/− DOCA and WT DOCA mice by comparison with untreated *Abcc6*−/− and WT mice. Interestingly, pro-mineralizing bone morphogenetic protein 4 (*Bmp4*) and periostin (*Postn*) gene expression significantly increased in *Abcc6*−/− DOCA compared to untreated *Abcc6*−/− but not in WT DOCA mice, reaching statistical significance subject to genotype (Fig. 5B). Finally, in the genes involved in matrix remodelling, expression of tissue inhibitor of metalloproteinase 1 (*Timp1*) was higher while matrix metalloproteinase 9 (*Mmp9*) was lower in both *Abcc6*−/− DOCA and WT DOCA mouse aortas than in untreated *Abcc6*−/− and WT mice. Matrix metalloproteinase 2 (*Mmp2*), plasminogen activator urokinase (*Plau*) and plasminogen activator inhibitor-1 (*Serpine1*) expression increased significantly in *Abcc6*−/− DOCA mice alone. Excepting *Timp2* and *Plat*, significant differences were recorded subject to genotype (Fig. 5C).

## Discussion

In the present study, we evidence that DOCA-salt-induced AHT gives rise to profound CV remodelling in *Abcc6*−/− mice which was associated with (i) calcium deposits in the heart characteristic of DCC; (ii) myocardial fibrosis; (iii) extensive vascular fibrosis and (iv) vascular gene expression involved in calcification and fibrosis.

This CV remodelling cannot be explained solely by a higher blood pressure since following DOCA-salt treatment, *Abcc6*−/− mice developed the same kinetic and amplitude of blood pressure rise (and the other hemodynamic parameters) as WT mice. This suggests that PXE patients may develop AHT as the general population and especially in salt-rich diet condition^14^.

Despite a similar gain in BP, only hypertensive *Abcc6*−/− mice developed myocardial calcifications. Progressive ectopic calcification is the hallmark of human and mouse PXE^2,15^. In mouse, such phenotype is evidenced by chronic age-related vibrissae calcifications^16^ or calcium deposits following acute cardiac damage, corresponding to DCC phenotype. DCC results from local tissue damage and cellular necrosis that occurs in infarcted tissue during myocardial healing^17^. To our knowledge, there has been no evidence of myocardial calcification following sustained hemodynamic changes in *Abcc6*−/− mice or PXE patients. These findings suggest that pressure overload imposed on the heart by increased peripheral arterial resistance could constitute a stress that contributes to induce dystrophic calcifications. Although DCC has not been reported in human PXE^18^, this phenotype may potentially predict the propensity of soft tissues to calcify when submitted to mechanical strain^19^. The fact that calcium deposits were not found by Von Kossa staining, a method routinely used on patient skin biopsies for diagnosis of PXE, may be explained by the patchy mineral deposition pattern in the LV of *Abcc6*−/− DOCA mice, as previously reported in the TA of old *Abcc6*−/− mice^20^.

In parallel to calcification, hypertensive *Abcc6*−/− mice developed a marked cardiac fibrosis.

There are very few data available on the incidence of CV fibrosis in PXE. Rau *et al*. proposed *Abcc6* as a candidate gene responsible of stress-induced cardiac fibrosis^21^. Moreover, Götting *et al*. demonstrated greater xylosyltransferase 1 (a marker of fibrosis) activity in the serum of PXE patients^22^. DOCA-salt model engenders CV remodelling that is representative of human volume-overload-induced AHT, involving vascular inflammation, hypertrophy, endothelial dysfunction and fibrosis^13^ Despite DOCA-salt model was reported to cause heart dysfunction witnessed by diastolic blood pressure (DBP) impairment^23^, we observed no differential effect of *Abcc6*−/− genotype on DBP or heart rate (HR). The absence of cardiac functional repercussion may be due to the lack of sensitivity of the method (tail-cuff plethysmography). Also, it is not excluded that investigation at later stage of hypertension would have given different results leading to myocardial dysfunction in the long term as previously reported in this model^23^. Of note, Abcc6 deficiency mitigates cardiac hypertrophy that commonly characterizes elevated BP. This contrasts with our previous study evidencing that old *Abcc6*−/− mice developed spontaneous heart hypertrophy but no fibrosis^24^. These data suggest that the pathological remodelling of the heart in the context of Abcc6 deficiency is different during ageing and hypertension.

Of note, we found increased fibrosis in the LV and TA of *Abcc6*−/− mice treated with DOCA-salt. Vascular fibrosis was associated with an increase of fibrosis-related genes regarding *Col3a1*, *Col1a1*, *Mmp2*, *Tgfβ2*, *Serpine1*, *Plau* and *Postn* in the aortic wall^25,26^. In human PXE fibroblasts, an upregulation of *TGFβ2* was reported^27^ although the exact nature of this dysregulation remains unclear^26^. MMP2 and MMP9 are thought to play a role in PXE, due to evidence of an increase in their serum levels in PXE patients^28^. From a cellular perspective, only differential expression of MMP2 could be demonstrated in PXE-fibroblasts^29,30^. In the present study, we found higher levels of *Mmp2* and *Timp1* expression in the TA of *Abcc6*−/− DOCA compared to untreated *Abcc6*−/− mice, likely indicative of ongoing ECM remodelling and increased collagen turnover in hypertensive *Abcc6*−/− mice^29^. Serpine1, also known as PAI-1 (Plasminogen activator inhibitor-1), is the principal inhibitor of tissue plasminogen activator (PLAT) and urokinase plasminogen activator (PLAU), and acts as a fibrinolysis inhibitor^31^. PLAU activity, converting plasminogen to plasmin, is directly controlled by Serpine1 itself modulated by TGFβ family members and contribute to PLAU dysregulation in various age-related diseases, demonstrating impaired tissue regeneration, inflammation, and fibrosis^32^. The findings from our study, revealed higher levels of *Serpine1* and *Plau* expression in the TA of hypertensive *Abcc6*−/− suggesting an exacerbated fibrotic process in *Abcc6*−/− DOCA mice. Periostin (Postn) is an ECM protein expressed predominantly by fibroblasts and plays an key role in cardiac hypertrophy and fibrosis following myocardial infarction^33^. We measured a greater expression of *Postn* in the TA of *Abcc6*−/− DOCA compared to untreated mice, evoking the link previously described between Postn and fibrotic response^34^. An increase in collagen type I synthesis and in the collagen type I/III ratio in vascular smooth muscle cells leads to matrix stiffness^35^. In the present study, we found higher levels of *Col1a1, Col3a1* expression in the TA of *Abcc6*−/− DOCA mice suggesting some remodelling associated with fibrosis and potentially underlying arterial ECM stiffening.

Another consequence of Abcc6 deficiency on vascular remodelling can be evidenced by a pro-calcifying genes expression pattern in the TA of hypertensive *Abcc6*−/− mice. There is a growing evidence that the involvement of TGFβ superfamily signalling pathway (mainly TGFβ and BMPs) and inorganic pyrophosphate (PPi) in PXE^26^, that may synergistically contribute to ectopic calcification. It has been shown that BMP signalling pathway is activated in hearts and aortas of *Abcc6*−/− mice^36^. Bmp4, a protein involved in vascular smooth muscle cell calcification^36^, was significantly higher in *Abcc6*−/− hypertensive mice than in WT mice. Interestingly, Bmp4 could be potentially instrumental in DCC following ischemia-reperfusion injury in *Abcc6*−/− mice^36^. We also evidenced a decrease in alkaline phosphatase (*Alp*) and an increase in ectonucleotidase (Enpp1) expression in WT DOCA mice, both favouring a reduction in Pi/PPi ratio preventing vascular calcification^37,38^. These changes were abrogated in *Abcc6*−/− mice, suggesting a lack of an anti-mineralization mechanism in these animals. Altogether, these results highlight a propensity for vascular calcification in *Abcc6*−/− mice treated with DOCA-salt. Medial arterial calcification is a hallmark of vascular aging^39^. Our results show that vascular calcification in hypertensive *Abcc6*−/− mice mimics what observed in older *Abcc6*−/− mice^20^. Hypertension accelerates age-related vascular remodelling and dysfunction^40^. Aging may impact the severity of vascular damage in hypertension^40^. Thus, close interactions between biological aging and the effect of high BP could exist^40^. The relevance of such specific remodelling pathways has yet to be validated in human PXE patients.

We can assume that such remodelling could lead in the long-term to the deterioration of cardiac and vascular function in *Abcc6*−/− mice and likely in PXE patients. Such hypothesis may be corroborated both in mouse and in human. Cardiac function of *Abcc6*−/− mice was previously assessed by our group. With the exception of a mild heart hypertrophy in the oldest pre-senescent animals, no major abnormalities could be evidenced in *Abcc6*−/− mice^24^. However, the situation may be different in pathological conditions. Unintuitively, the DOCA-salt-induced hypertrophic remodelling was limited both in LV and aorta of *Abcc6*−/− mice. No significant correlation between heart or TA hypertrophy and the fibrotic score could be evidenced neither in *Abcc6*−/− nor in WT mice DOCA (not shown). Although speculative, the conclusion may be that early fibrosis (or calcification) would have blunt the hypertrophic process.

Vascular function investigations of medial thoracic aorta revealed that despite a gain of contractile response to serotonin (and phenylephrine), DOCA-salt treatment did not impair endothelial function neither in WT nor in *Abcc6*−/− mice. However, we cannot exclude that an endothelial dysfunction would have been assessed in resistance arteries of hypertensive DOCA-salt mice as it has been previously reported^41^. Such apparent discrepancy with our findings may result from the difference between resistance and compliance vessels in the context of a DOCA-salt regimen^41^.

In our previous study conducted on both resistant and conductance arteries of WT and *Abcc6*−/− mice, no differences were found in endothelial dependent and -independent relaxation as well as phenylephrine and thromboxane analogue-dependent contraction^20^. Here, in pathological conditions, the vascular impact of Abcc6 deficiency was still not revealed functionally. This may be explained by the fact that fragments used for vascular reactivity (medial thoracic aorta) were different from those used for histological analysis which was more proximal (adjoining aortic arch) and prone to fibrosis/calcification^20^. Also, histological quantification of fibrosis using Sirius red is semi quantitative. Although the staining was corroborated by a molecular approach it is difficult to evaluate the real extent of fibrosis and its functional impact. Absence of endothelial dysfunction in hypertensive *Abcc6*−/− mice may nevertheless suggest that PXE-associated PAD contrasts with other calcific metabolic diseases such as diabetes, chronic renal insufficiency and atherosclerosis, which incur early onset of endothelial dysfunction^42^. To our knowledge, no incidence of endothelial dysfunction or flow-mediated dilation has been reported in PXE patients. With reference to contractile response, our results are coherent with previous data showing that loss of *Abcc6* had a limited impact on receptor-dependent arterial contraction, while it was associated with increased myogenic tone^20^.

To our knowledge, there is no data reporting CV remodelling of *Abcc6*−/− mice. Extensive fibrosis and worsening of calcifications were not expected and raised the question of the therapeutic potential of anti-hypertensive drugs. Different treatments targeting renin angiotensin system or endothelin receptors^43–45^ have been shown to potently reduce cardiac fibrosis in the DOCA-salt model without affecting BP rise while sympatholytic such as hydralazine reduces BP with however incomplete effect on fibrosis^46^, evidencing the dissociation between efficacy of pharmacological treatment to prevent fibrosis and to blunt BP. From our findings, we can assume that calcifications and myocardial fibrosis in DOCA-salt mice could be independent on elevated blood pressure. Based on the studies cited above, it would be interesting to see whether antihypertensive treatments would work efficiently to reduce fibrosis in *Abcc6*−/− mice and whether these treatments could also improve calcification, which constitutes a specificity of this model.

Indeed, in a model of heart failure^21^, *Abcc6*−/− mice were shown to develop heart fibrosis and calcification following myocardial infarction or necrotic heart lesions^47^. However, in these studies the cardiac function was not investigated. Hence, the functional repercussion of this CV remodelling remains questioned, in these pathological situations as well as during hypertension. In human PXE patients, Campens *et al*. reported heart and vascular dysfunction including increased pulse wave velocity and altered left ventricular diastolic function^11^. The first most likely reveals arterial stiffness due to excessive calcium deposit in the arterial wall as previously reported in *Abcc6*−/− mice^20^ and PXE patients^8,11,20^. We believe that the second may be linked to structural (fibrosis and/or calcification?) alterations of the myocardium.

Vascular stiffness has been reported in *Abcc6*−/−^20^ mice and PXE patients^8,11^. Cardiovascular remodelling in PXE is an important question, especially in the context of common pathological condition such as hypertension. We think that the calcific fibrotic remodelling we evidence here may originate long term CV dysfunction in agreement with the clinical feature of PXE patients. The investigation of the cardiac function of hypertensive *Abcc6*−/− mice as well as the potential protective effect of anti-hypertensive molecules would deserve specific attention.

## Materials and Methods

### Ethical standards

The data that support the findings of this study are available from the corresponding author upon reasonable request. The corresponding author had full access to all the data in the study and takes responsibility for its integrity and the data analysis. The lead author wrote the first draft of the manuscript, and all co-authors participated in and approved subsequent revisions.

### Animals

The investigation was conducted in accordance with guidelines from Directive 2010/63/EU of the European Parliament on the protection of animals used for scientific purposes (authorization of the laboratory 849007003). The protocol was approved by the Institutional Animal Care and Use Committee (IACUC): Committee on the Ethics of Animal Experiments (CEAA) of “Pays de la Loire” (permits CEEA.Pdl n°06, and APAFIS#4570-20l6031716512454 v3 from the “Ministère de l’Education Nationale, de l’Enseignement Supérieur et de la Recherche”). *Abcc6*−/− deficient mice (*Abcc6*^*tm1Aabb*^ hereafter referred to as *Abcc6*−/− mice) were generated as previously described^12^. Mice were backcrossed to a C57BL/6 J background more than 10 times, and reproduction was obtained using heterozygous breeders. Adult male mice aged 23 to 50 weeks were used to avoid bias arising from age-related arterial stiffness.

### Models of hypertension

*Abcc6*−/− and WT mice were unilaterally nephrectomised under isoflurane inhalation anaesthesia (2.2–2.6% isoflurane in 100% O_2_). Hind-paw withdrawal, blink reflex and respiratory rate were monitored in these mice. A 1.5-cm incision was made through the skin and abdominal muscle caudal to the rib cage. The renal artery and vein were ligated with 4-0 silk sutures (Ethicon) and the left kidney was removed. Skin and muscle layers were closed separately with 4-0 silk sutures. In addition, a 1-cm incision was made in the back, at the base of the neck to implant DOCA pellets subcutaneously (Innovative Research of America, Sarasota, Florida, USA), to provide a dose of 1 mg/kg/day. Mice were then administered water containing 0.9% NaCl and 0.2% KCl. Upon recovery, they were monitored each day for 19 days to measure SBP, DBP and HR using tail-cuff plethysmography^48^.

### *Ex Vivo* Pharmacological investigation

Mice were sacrificed at day 19 after DOCA pellet implantation. Immediately after euthanasia, TA arteries were dissected in ice-cold physiological saline solution (PSS: mmol/L 130.0, NaCl; 15.0, NaHCO3; 3.7, KCl; 1.6, CaCl2; 1.2, MgSO4 and 11.0, glucose). Pharmacological investigation was conducted on 2 mm-long arterial segments mounted on a wire-myograph (DMT, Aarhus, DK). Initial contraction to potassium chloride (KCl 80 mM), and cumulative concentration-dependent contraction in response to serotonin (5-HT) was tested in resting arteries. Endothelium-dependent and -independent relaxation were measured using ACh and nitric oxide donor (SNP), respectively. Relaxing responses, measured by cumulative concentration-response curves, were established on phenylephrine-contracted (1 μM) arteries.

### Calcium quantification

Calcium quantification in the LV of the heart was determined using colorimetric analysis^49^. One half of each murine LV was weighed, dried and incubated at room temperature for 48 hours in 0.15 N HCl. These samples were then centrifuged at 15,000 × g, and total calcium content of the supernatant was calculated using the Calcium Liquicolor Test Kit (Stanbio, Boerne, TX). The results were normalized to tissue weight and values were expressed in mg/g dry tissue.

### Pathological analysis

The remaining half of each murine LV and TA tissues were embedded in OCT (Optimal Cutting Temperature) compound. Von Kossa staining to determine calcium deposits and Sirius red staining to determine type I and III collagen fibers were undertaken on 5μm-thick frozen sections. Tissue sections were analyzed under an Olympus AX70 microscope (Olympus, Tokyo, Japan). Quantification of red-stained fibrotic areas was obtained from five different fields for each tissue sample at 400x magnification (corresponding to a field size of 0.344-mm^2^), using Cell-F software and a ColorView III camera (Olympus, Tokyo, Japan). Results were expressed as the mean value of the five different field findings from each tissue sample. Aorta thickness was measured in 5 μm-thick frozen sections using Image J software (National Institutes of health NIH, USA) (three to six aorta sections per animal).

### RT-qPCR

Gene expression was investigated using quantitative polymerase chain reaction after reverse transcription of total RNA (RT-qPCR). Segments of dissected TA were stored at −20 °C in RNAlater Stabilization Reagent (Qiagen, Valencia, CA, USA) until use. RNA was extracted using the RNeasy^®^ Micro Kit (Qiagen, Valencia, CA, USA) as per manufacturer instructions. RNA extracted from these aortas (200 ng) was used to synthesize cDNA using the QuantiTect^®^ Reverse Transcription Kit (Qiagen, Valencia, CA, USA). RT-qPCR was performed with Sybr^®^ Select Master Mix (Applied Biosystems Inc., Lincoln, CA, USA) reagent using a LightCycler 480 Real-Time PCR System (Roche, Branchburg, NJ, USA). Primer sequences are shown in Supplementary Table S1. *Gapdh, Hprt and Gusb* were used as housekeeping genes. Analysis was not performed when Ct values exceeded 35. Results were expressed as: 2^(Ct target-Ct housekeeping gene)^.

### Data analysis and statistics

Results were expressed as mean ± SEM. Statistical analysis was performed using GraphPad Prism software (La Jolla, CA, USA). The respective roles of DOCA-salt treatment and genotype were tested by means of a 2-way ANOVA test. Where interaction between the 2 factors was observed, the effect of DOCA-salt treatment was studied in each genotype using the Bonferroni post-hoc test. 2-way repeated measures ANOVA was used to analyse hypertension and *ex vivo* pharmacological findings. Vascular gene expression data were compared using the Student’s t-test. A p-value <0.05 was considered statistically significant. For genes expression, the statistical analysis was performed on raw data to evaluate (i) the effect of hypertension in both genotype separately (ii) the effect of the genotype in control (non-hypertensive) mice and (iii) the effect of the genotype in hypertensive mice. For sake of readability results were shown as fold change in gene expression levels compared to untreated mice.

### Translational perspective

**Figure 6:**
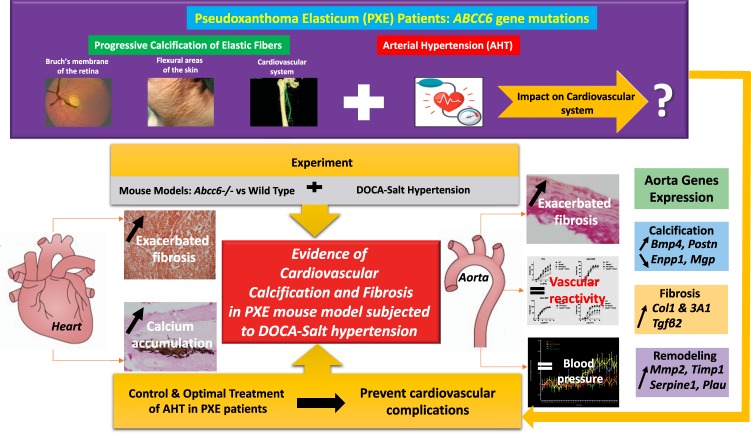
General synthesis of the study suggesting an evidence of cardiovascular calcification and fibrosis in Pseudoxanthoma Elasticum Mouse Models Subjected to DOCA-Salt Hypertension.

The present study demonstrates that *Abcc6* loss-of-function is associated with CV calcification and fibrosis following chronic AHT (Fig. 6). Hypertension leads to a vicious circle of pathological vascular remodelling including thickness, stiffness, fibrosis, calcification and aging^25,40^. Thus CV aging may be responsible for high residual lifetime risk for developing AHT in middle-aged and elderly individuals^50^. Our findings suggest accelerated pathological CV remodelling in hypertensive *Abcc6*−/− mice leading to fibrosis and calcification in heart and aorta independent of atherosclerosis, inflammation and thrombosis as detected in PXE patients^51^. Finally, the current treatment approach for slowing or minimizing CV manifestations of PXE is based on reducing CV risk factors through lifestyle changes (smoking cessation, weight loss, moderate physical exercise)^18^. There are no specific recommendations for AHT management in PXE patients since no randomized trial has investigated this issue to date. While arterial thickness and stiffness are increased in PXE patients^8^ regardless of BP and given the impact of AHT on thickness and stiffness^50^, our results pinpoint the importance of diligent monitoring of BP in PXE patients, in accordance with the Blood Pressure Treatment Guideline Recommendations and Cardiovascular Risk^52^. Choice of BP medication should target central arterial pressure to prevent aortic thickness and stiffness as demonstrated in the Conduit Artery Function Evaluation (CAFE) study^53^.

### Ethics approval and participant consent

All procedures described in this report have received ethics committee approval.

## Conclusion

This study aimed at assessing the interplay between hypertension and PXE. While CV calcifications is a hallmark of PXE, Abcc6 deficiency allowed extensive fibrosis to occur in mice subjected to DOCA-Salt hypertension. Perturbations in the expression of genes implicated in the synthesis, organization, and degradation of ECM co-occurred with the activation of osteogenic signalling in PXE mice. Future investigations should be designed to characterize cardiac and vascular molecular signature of this abnormal remodelling in order to orientate on proper therapies to prevent hypertension-associated CV complications in PXE.

## Supplementary information

Table S1
