# Supplementary material for: Evidence of Cardiovascular Calcification and Fibrosis in Pseudoxanthoma Elasticum Mouse Models Subjected to DOCA-Salt Hypertension
Source: Sci Rep. 2019 Nov 8;9:16327. doi: 10.1038/s41598-019-52808-z (PMC6841718; doi:10.1038/s41598-019-52808-z)
Supplement: Supplementary file 1 — Table S1 [file 41598_2019_52808_MOESM1_ESM.docx]

**Evidence of Cardiovascular Calcification and Fibrosis in Pseudoxanthoma Elasticum Mouse Models Subjected to DOCA-Salt Hypertension**

Loukman OMARJEE MD-PhD^a,b,c,d,e^; Charlotte ROY PhD^a^; Christophe LEBOEUF PhD^f,g^; Julie FAVRE, PhD^a^, Daniel HENRION PhD^a^; Guillaume MAHE MD-PhD^b,c^; Georges LEFTHERIOTIS MD-PhD^h^; Ludovic MARTIN MD-PhD^a,d^ , Anne JANIN MD-PhD^f,g,i^; Gilles KAUFFENSTEIN PhD^a,d^

^a^ MitoVasc Institute, UMR CNRS 6015 - INSERM U1083, Angers University, France

^b^ Univ Rennes, CHU Rennes, INSERM CIC1414, Vascular Medicine Unit, Rennes, France

^c^ PXE Vascular Consultation Centre, CHU Rennes, 35000 Rennes, France

^d^ PXE Reference Centre (MAGEC Nord), University Hospital of Angers, Angers, France

^e^ Vascular Medicine Unit, Redon Hospital, 8 Rue Etienne Gascon, 35600 Redon, France

^f^ Pathology Laboratory, Paris Diderot University, Sorbonne Paris Cité, Paris, France

^g^ INSERM U942, Paris, France

^h^ Department of Physiology and Vascular Investigation, Nice University Hospital, France

^i^ Department of Pathology, Saint-Louis Hospital, APHP, Paris, France

Correspondance:

**Dr Gilles KAUFFENSTEIN PhD**

UMR INSERM 1260 - Nanomédecine Régénérative

Université de Strasbourg - Faculté de Pharmacie

74, route du Rhin - BP 60024

FR-67401 Illkirch-Graffenstaden

Email : kauffenstein@unistra.fr

**Dr Loukman OMARJEE, MD-PhD**

Vascular Physician and Clinical Immunologist

PXE Vascular Consultation Centre

Department of Vascular Medicine and Investigation

Hôpital Pontchaillou – Rennes University Hospital

2 rue Henri Le Guilloux Rennes, F-35033. France.

Contact Phone Number: +33 (0) 2 99 28 43 21

Contact Mobile Number: +33 (0) 6 27 49 70 51

Contact Email: [loukmano@yahoo.fr](mailto:loukmano@yahoo.fr) Twitter: @LoukmanOmarjee

**Table S1.** Primer sequences. Forward and reverse primer sequences of genes used for RT-qPCR showing position, amplicon size and intron. *Gapdh, Hprt and Gusb* were used as housekeeping genes.
